# Posology and Serum-/Xeno-Free Engineered Adipose Stromal Cells Cell Sheets

**DOI:** 10.3389/fcell.2022.873603

**Published:** 2022-04-26

**Authors:** Jun Ochiai, Larakaye Villanueva, Hope Niihara, Yutaka Niihara, Joan Oliva

**Affiliations:** Emmaus Life Sciences, Inc., Torrance, CA, United States

**Keywords:** mesenchymal stem cells, cell sheets, transmittance, multilayer, serum-/xeno free, posology

## Abstract

Well-characterized adipose stem cells and chemically defined culture media are important factors that control the production of the cell sheet, used in translational medicine. In this study, we have developed and engineered multilayer adipose stem cell cell sheets (ASCCSs) using chemically defined/serum-free culture media: undifferentiated or differentiated into osteoblasts and chondrocytes. In addition, using the cell sheet transmittance, we estimated the number of cells per cell sheet. Undifferentiated ASCCSs were engineered in 10 days, using serum-free/xeno-free culture media. They were CD29^+^, CD73^+^, CD90^+^, CD105+, HLA-A+, and HLA-DR-. ASCCSs differentiated into chondrocytes and osteoblasts were also engineered using chemically defined and animal-free culture media, in only 14 days. The addition of an ROCK inhibitor improved the chondrocyte cell sheet engineering. The decrease in the cell sheet transmittance rate was higher for the osteoblast cell sheets due to the intracellular Ca^2+^ accumulation. The estimation of cell number per cell sheet was carried out with the transmittance, which will provide important information for cell sheet posology. In conclusion, three types of ASCCSs were engineered using serum-free, xeno-free culture media, expressing their specific markers. Their transmittance measurement allowed estimating the number of cells per cell sheet, with a non-invasive methodology.

## Introduction

In 2006, a consortium of experts released the minima criteria of mesenchymal stem cells (MSCs): adherence to a plastic surface, expression of specific MSC markers (CD73^+^, CD90^+^, CD105^+^, CD11b^−^, CD14^−^, CD34^-^, CD45^−^, and HLA-DR^-^) and the capacity to differentiate into cells from the three embryonic germ layers: ectoderm, mesoderm, and endoderm (Dominici et al., 2006). The International Society for Stem Cell Research, gathering experts in the field of cell and gene therapy, already released guidelines in 2006 and updated ones in 2018, based on two decades of experience (ISSCR, 2016). The use of MSCs offers different advantages such as unlimited and easy availability and low immunoreactivity. The interest in using mesenchymal stem cells for translational application keeps increasing since the discovery of different sources of MSCs: umbilical cords, adipose tissues, dental pulp, and peripheral blood (Nagamura-Inoue and He, 2014). In January 2021, 1,259 clinical trials were found using mesenchymal stem cells (www.clinicaltrials.gov). Clinical trials involving MSCs are currently targeting a wide range of diseases such as cardiac disease, diabetic nephropathy, autoimmune diseases, and liver failure. Because of the increase of conducted clinical trials and the experience acquired from these trials, federal agencies have modified and improved their guidelines for cell and gene therapy to ensure the patient’s safety and create new regulations to adapt to the new cell and gene therapy clinical trials (Konomi et al., 2015; Administration FDA, 2020b).

Isolated MSCs require not only their characterization but also their quality must be ensured after their expansion and/or differentiation because billions of cells can be required in clinical trials. It is recommended by the FDA that cell-based therapeutics developed in laboratories should be very similar to the products used in translational applications. Results obtained in laboratories using the products for research only can be very different from the cell-based therapeutics developed with United States Pharmacopeia (USP)–grade compounds and with master and working cell banks. Several manufacturers ensure that good quality MSCs are being provided to laboratories for their research and have adapted their production and tests based on FDA guidelines. Animal serum has always been a concern for the FDA because of the potential transmission of bovine spongiform encephalopathy (Administration FDA, 2020a). In addition to the potential infection from animal-contaminated animal serum, the composition of animal serum varies between each lot, making it difficult to obtain reproducible results in laboratories. Many laboratories have explored different sources of serum or supplements for cell culture to use as an alternative for animal serum. For example, epithelial cell sheets are engineered using mice 3T3 NIH feeder cells in the presence of fetal bovine serum (FBS), since the 1970s (Rheinwald and Green, 1977). To address the concerns of regulatory agencies, different laboratories have engineered epithelial cell sheets by eliminating the need for xeno feeder cells and FBS. In 2009, xeno feeder cells were replaced by bone marrow stem cells to engineer epithelial cell sheets, but FBS was still used (Omoto et al., 2009). Okano’s group stepped forward and engineered cell sheets by using human autologous serum, in absence of feeder cells and animal serum (Murakami et al., 2006). However, the variability of the serum between the patients could be an influencing factor in the success rate of cell-sheet engineering and will require additional tests to characterize the patient serum (Murakami et al., 2006). Other groups used human platelet lysate to replace the animal or human serum (Becherucci et al., 2018). In terms of ASC proliferation, Gimble’s group showed that 0.75% of the platelet lysate is equivalent to 10% FBS, and the platelet lysate maintained the differentiation properties of the ASCs (Cowper et al., 2019). However, another group showed that 5% of platelet lysate is the equivalent of 10% FBS in terms of the proliferation of ASCs (Kakudo et al., 2019). The quality of cells and/or serum substitutes could be the factors explaining the divergences in the results, underlying the importance of controlling the quality of every product used in cell culture. Manufacturers also developed culture media compatible with human trials, based on the changes and requirements in federal agency guidelines. Chemically defined, xeno-/serum-free culture media to maintain MSC properties are an important criterion to reproduce the results and compare the outcomes. Chemically defined serum-free culture media is the best option to control the proliferation and differentiation of the cells and compare the outcome of treated patients. Many publications report the use of serum-free culture media to expand and differentiate ASCs (Chase et al., 2010) and showed better results. The differentiation of ASCs into dopaminergic neurons was higher in serum-free culture media than in culture media with a low animal serum percentage. In addition, differentiated cells, with serum in the culture media, secreted twice the amount of tyrosine hydroxylase (Faghih et al., 2019).

Cell sheets have advantages over the injection of isolated cells such as a higher survival rate of the injected cells, accurately targeting the damaged area, and therefore better healing properties (Gyongyosi et al., 2008; Urban et al., 2008; Hamdi et al., 2011; Katagiri et al., 2013). In the United States, only hematopoietic progenitor cells derived from umbilical cord blood are used to treat patients with blood production disorders, and no MSC product has been approved by the FDA while they are used in clinical trials. In many of the clinical trials, the MSCs are injected in the patients. It is an easy and cheap methodology to treat patients, and the posology can be well-controlled. The healing mechanism of action of the MSC is not yet well-determined. It could be done through the physical cell-to-cell interaction or it could be because of the paracrine effect (Xiang et al., 2009; Spees et al., 2016; Jiang et al., 2017), which is becoming a more popular theory. However, injected cells can migrate ectopically in the organism, mainly in the lungs (Pereira et al., 1998; Gao et al., 2001; Schrepfer et al., 2007; Lee et al., 2009), which could lead to health issues such as thrombosis, impairment of the organs, or tumor formation (Lee and Hong, 2017; Coppin et al., 2019).

Our team has previously reported successful engineering of human adipose stem cell cell sheets (ASCCSs), using culture media complete with serum (Oliva et al., 2019). In this study, we propose to engineer three different types of ASCCSs with chemically defined culture media (xeno- and serum-free culture media): undifferentiated, chondrocyte, and osteoblast multilayered cell sheets. In addition, using a transmittance device developed by our group, the difference in transmittance was studied to establish standard curves of the ASCCSs during their differentiation (Ochiai et al., 2021). We also estimated the number of cells per cell sheet using the transmittance values, which is important information to provide to federal agencies in terms of posology.

## Materials and Methods

### Cell Culture

Human adipose stromal cells (ASCs) were purchased from RoosterBio, Inc (RoosterBio, Inc., Frederick, MD). Human ASCs were used for the following experiments. The hASCs were expanded up to passage 5, in a T75 flask (USAScientific, Ocala, FL), using RoosterNourish™-MSC-XF (RoosterBio, Inc., Frederick, MD).

### Engineering of the Adipose Stromal Cell Cell Sheets

ASCs were seeded at 10.2 × 10^4^ ASC per cm^2^ in a 35-mm culture dish (Corning, Corning, NY). The ASCs were cultured with RoosterNourish™-MSC-XF culture media (RoosterBio, Inc., Ballenger Creek, Maryland). The differentiation of the cell sheets started when they reached confluence, on day 4 after initial seeding.

- Undifferentiated ASCCSs: ASCs were cultured with RoosterNourish™-MSC-XF. The culture media were replaced every 2 days, up to 18 days from the initial seeding day.
- Osteoblast cell sheets: ASCs were cultured with Osteomax-XF Differentiation Medium (Millipore-Sigma, Burlington, MA). The culture media were replaced every 3 days, up to 17 days from the initial seeding day.
- Chondrocyte cell sheets: ASCs were cultured with a MesenCult™-ACF Chondrogenic Differentiation Kit (Stem Cell, Vancouver, Canada). The culture media were replaced every 2 days, up to 19 days from the initial seeding, and were also replaced when 10 M of ROCK Inhibitor y-27632 (MedChemExpress, Monmouth Junction, NJ, United States) was used.
- Skeletal muscle differentiation medium (PromoCell, Heidelberg, Germany) was used to show that the cells do not form a multilayer cell sheet and the cell density does not change by measuring the transmittance.

### Dyeing of the Adipose Stem Cells Cell Sheets

Multilayer undifferentiated, chondrocyte and osteoblast cell sheets cells were stained with the Alcian Blue Stain kit (Bioquochem, Llanera, Spain) and with the Alizarin Red Stain kit (Millipore-Sigma, Burlington, MA). The protocols from the manufacturer were modified to stain the cross section of the cell sheets.

For Alcian blue, fixed tissues are deparaffinized with xylene treatment (Fisher Scientific, Waltham, MA, United States) and by hydrating them with 100 (two times), 90, and 50% ethanol (Millipore-Sigma, Burlington, MA). The tissues are washed with deionized water and washed 2 × 1 min with 3% glacial acetic acid. Alcian blue is added on top of the tissue for 30 min at room temperature. Alcian blue will be rinsed with 3% glacial acetic acid (Bioquochem, Llanera, Spain). The tissues are washed with tap water (1 min) and deionized water (1 min), and the washing steps are repeated. The nuclei are dyed with nuclear fast red for 5 min. The tissues are washed with tap water (1 min) and deionized water (1 min), and the washing steps are repeated. The tissues are dehydrated with 70, 90, and 100% ethanol. Ethanol is washed three times with xylene. Mounting media (Alban Scientific, St Louis, MO) and coverslips (Fisher Scientific, Waltham, MA, United States) are placed on the tissues.

For Alizarin Red, the fixed tissues are deparaffinized with xylene treatment and by hydrating them with 100 (two times), 90, and 50% ethanol. The tissues are washed with deionized water for 2 × 5 min. Alizarin Red is added on top of the tissue for a few minutes at room temperature. Alizarin red is removed with tap water (1 min) and deionized water (1 min), and the washing steps are repeated. The tissues are dehydrated with 70, 90, and 100% ethanol. The tissues are then rinsed with acetone, acetone: xylene (1:1) (Millipore-Sigma, Burlington, MA) followed by washing thrice with xylene. Mounting media and coverslips are placed on the tissues.

### Transmittance of the Differentiated or Not Differentiated ASCCSs

The transmittance is measured only after the culture media are replaced, and it was measured only when the cells reached confluence until they form a multilayer cell sheet. The transmittance of the adipose stromal cells cell sheets was measured using the device described in the reference (Ochiai et al., 2021). In summary, the cell culture dish is placed on the stage, always in the same position. The edge of the cell culture dishes is marked to ensure that the cell culture dishes are always placed in the same position to measure the transmittance of the same nine spots. A light, produced by the light source, will shine through the cell sheet, and the light intensity will be measured by a detector placed under the stage. A single measurement is an average of 100 values per read from the detector with 10 reads per second, for 10 s per point (nine points per cell sheet) and per cell sheet (Ochiai et al., 2021). The reference value was obtained by measuring the intensity of the light coming through a cell culture dish that has the same amount of culture media but with no cell sheet (blank sample). The collected values were converted to percent transmittance with respect to the value of the reference blank sample.

### Harvesting of the ASCCSs

To harvest the multilayer cell sheets, we used a CellShifter membrane, with a 30 mm diameter (CellSeed, Inc., Tokyo, Japan) and forceps. The forceps (Roboz, Gaithersburg, MD) were used to cut the edge of the cell sheet from the cell culture dish wall. The CellShifter was placed on top of the cell sheet. The culture media were removed, and the side of the cell sheet was wrapped over the edge of the CellShifter. By using forceps, the cell sheet was lifted and placed in a new cell culture dish. Once placed in a cell culture dish, a few drops of PBS were poured over the CellShifter. Using the forceps, the CellShifter was lifted and separated from the cell sheet, which stayed attached to the cell culture surface. The cell sheets were fixed in 10% neutral buffered formalin for immunohistochemistry staining (Fisher Scientific, Waltham, MA, United States) or entirely used for measuring the DNA content. It is important to report that it was not possible to harvest ASCCSs, cultured with skeletal muscle differentiation culture media, because the cells did not form a multilayer cell sheet.

### Estimation of Cell Numbers per Cell Sheet

The number of cells was estimated by isolating the total genomic DNA from the entire cell sheets and compared with the quantity of genomic DNA from a determined number of isolated ASCs. Genomic DNA, from isolated ASCs and multilayer cell sheets, were isolated with the Wizard Genomic DNA Purification Kit (Promega, Madison, WI, United States), following the manufacturer’s protocol. After harvesting the cell sheets, the ASCCSs were dissociated by sonication (Fisherbrand™ Model 705 Sonic Dismembrator) at 20 Hz for 5 s (Fisher Scientific, Waltham, MA, United States). The quantity of double-stranded DNA per cell was estimated using the QuantiFluor dsDNA System (Promega, Madison, WI, United States), using an aliquot of the total DNA. The standard curve shown in Figure 6A and the dilution factor of the aliquot were used to estimate the number of cells per cell sheet.

### Immunocytochemistry Staining

The engineered cell sheets were fixed in 10% neutral buffered formalin and embedded in paraffin. The tissue sections were used for immunofluorescent staining with CD19 (Cat # NBP2-25196), CD73 (Cat # NBP2-25237) (NovusBIo, CO, United States), CD29 (Cat # ab134179), HLA-A (Cat # ab52922), HLA-DR (Cat # ab92511) (Abcam, MA, United States), and Oct3/4 (Cat # NB-100-2379SS) (Novus Biologicals LLC., Littleton, CO, United States). Alexa Fluor 488 donkey anti-rabbit conjugated secondary antibodies and Alexa Fluor 488 donkey anti-mouse conjugated secondary antibodies (Invitrogen, Carlsbad, CA, United States) were used. Propidium iodide (Invitrogen, Carlsbad, CA, United States) was used to stain nuclear DNA. An EVOS M5000 microscope was used to analyze the slides (Invitrogen, Carlsbad, CA, United States).

### Statistical Analysis

All results are expressed as the mean ± standard deviation. The data were analyzed using one-way ANOVA. A *p* < 0.05 was considered statistically significant.

## Results

**FIGURE 1:**
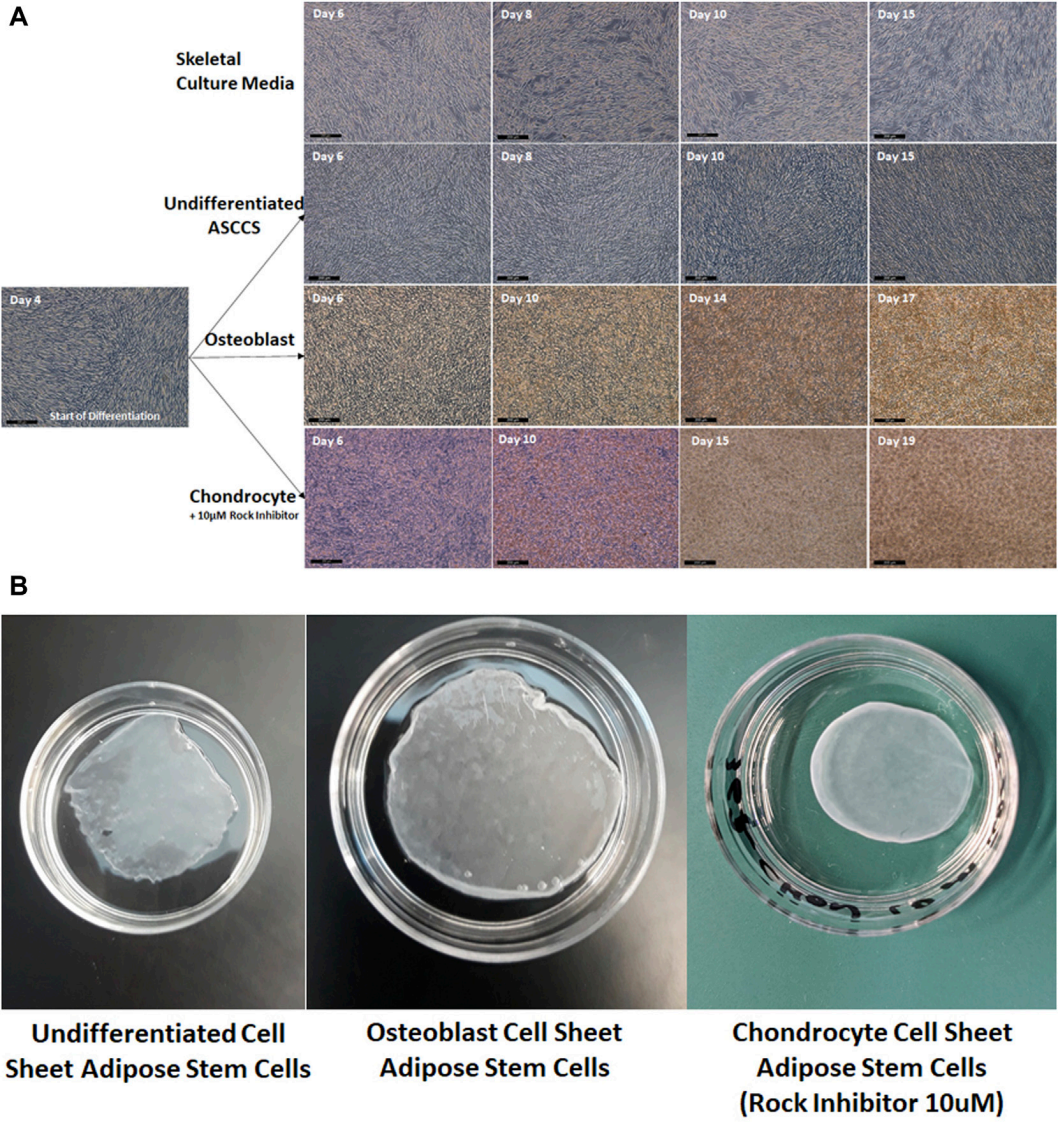
**(A)** Pictures of the cell sheets, during the differentiation time (the scale bars are 200 µm). **(B)** Pictures of the harvested cell sheets, placed in 35-mm cell culture dishes.

In Figure 1, pictures of the cell sheet formation, over time, show the increase of the cell densities. On day 4, when the ASCs reached confluence, the culture media were replaced. As a negative control, the ASCs did not form a multilayer cell sheet when they were cultured with skeletal culture media (used to differentiate iPSCs into myoblast) (Figure 1A). The cell density did not increase, which is related to the level of transmittance maintained at around 80%, up to 16 days (Figure 3). The ASCs kept growing over time, forming a strong multilayer cell sheet (Figure 1A). The transmittance of these undifferentiated ASCCSs decreased at around 60% as reported in a previous publication but using different culture media (Ochiai et al., 2021). Osteoblast cell sheets were engineered in 15 days. The ASCs differentiated very fast, in a few days, and differentiation increased over time. This can be noticed from the orange deposit at the top of the cell sheets (Figure 1A).

On day 4, the ASCCSs were treated with the MesenCult™-ACF Chondrogenic Differentiation Kit, but the success rate of the engineered chondrocyte cell sheets was very low. Very few chondrocytes cell sheets were engineered in 25 days. The cell sheets were detaching spontaneously during differentiation (data not shown). We noticed that the cell sheets always detached from the edges due to centripetal forces. To overcome the spontaneous detachment, a ROCK inhibitor (10 µM) was used during the differentiation period. The use of the ROCK inhibitor had two advantages: 1) Cell sheets never detached during the differentiation period and 2) the time for differentiation was shortened by around six days (Figure 1A).

In Figure 1B, the harvested cell sheets are shown. It is difficult to describe the physical properties of the cell sheets when harvested, but it is important to mention that the chondrocyte cell sheets have a rubber physical property when they were touched with forceps. This physical property plays a role in maintaining the round shape of the cell sheet. Osteoblast cell sheets were more rigid when they were touched with forceps.

**FIGURE 2:**
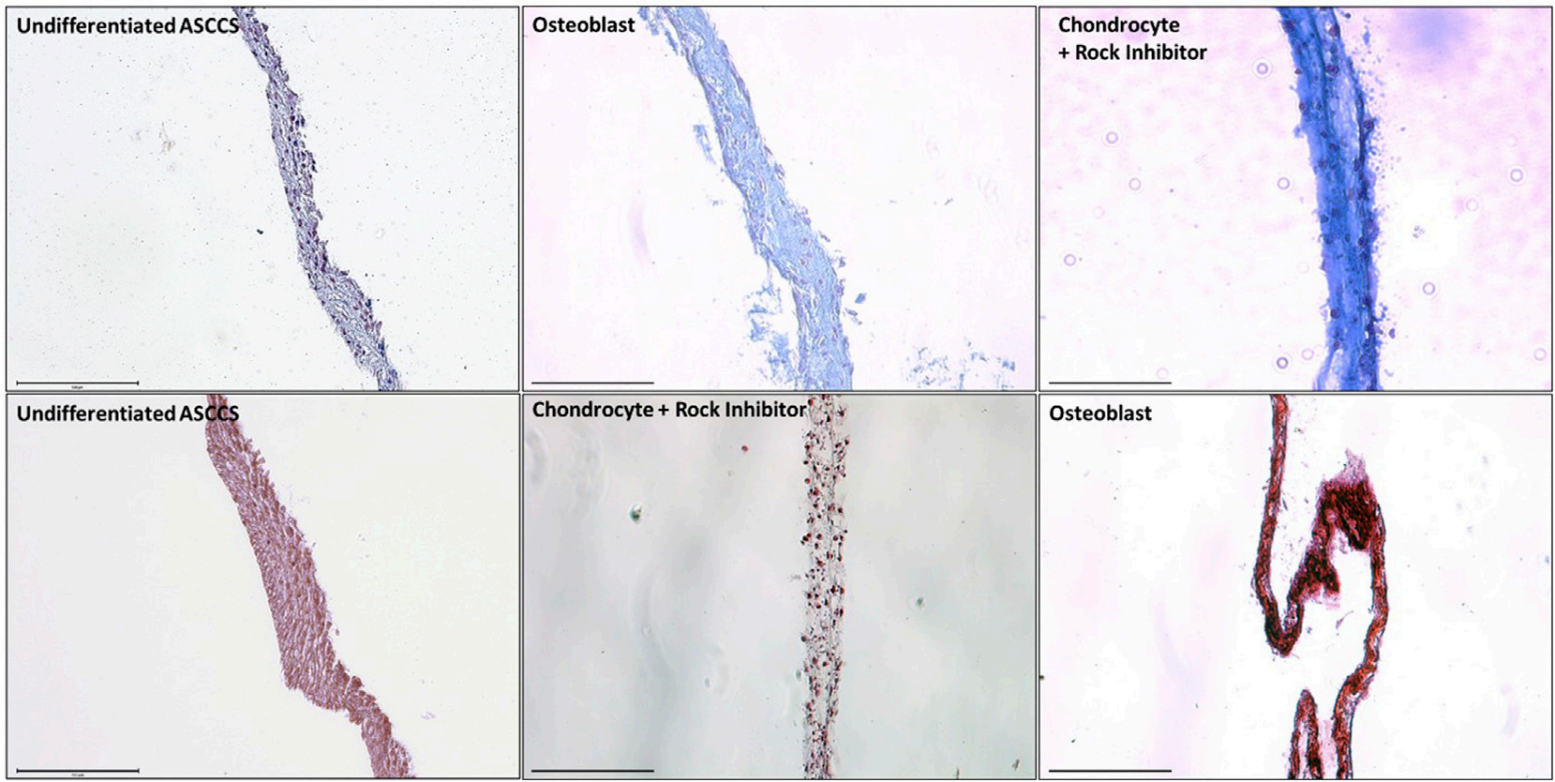
Dye of the undifferentiated ASCCSs, chondrocyte cell sheets, and osteoblast cell sheets with Alcian blue and Alizarin red. Scale bars are 150 µm.

In Figure 2, the cell sheets were dyed with Alcian blue and Alizarin red to show the level of differentiation. Alcian blue dyes strongly to the glycosaminoglycans, which are present on the cartilage (Ovchinnikov, 2009; Rigueur and Lyons, 2014). Alcian blue stained more differentiated chondrocyte cell sheets than the undifferentiated ASCCSs or the osteoblast cell sheets (Figure 2 top row). Alizarin red binds to calcium, which is one of the major ions present in the bones (Zhu and Prince, 2012). Alizarin red strongly dyed the osteoblast cell sheets compared with the undifferentiated ASCCSs or the chondrocyte cell sheets (Figure 2 bottom row).

**FIGURE 3:**
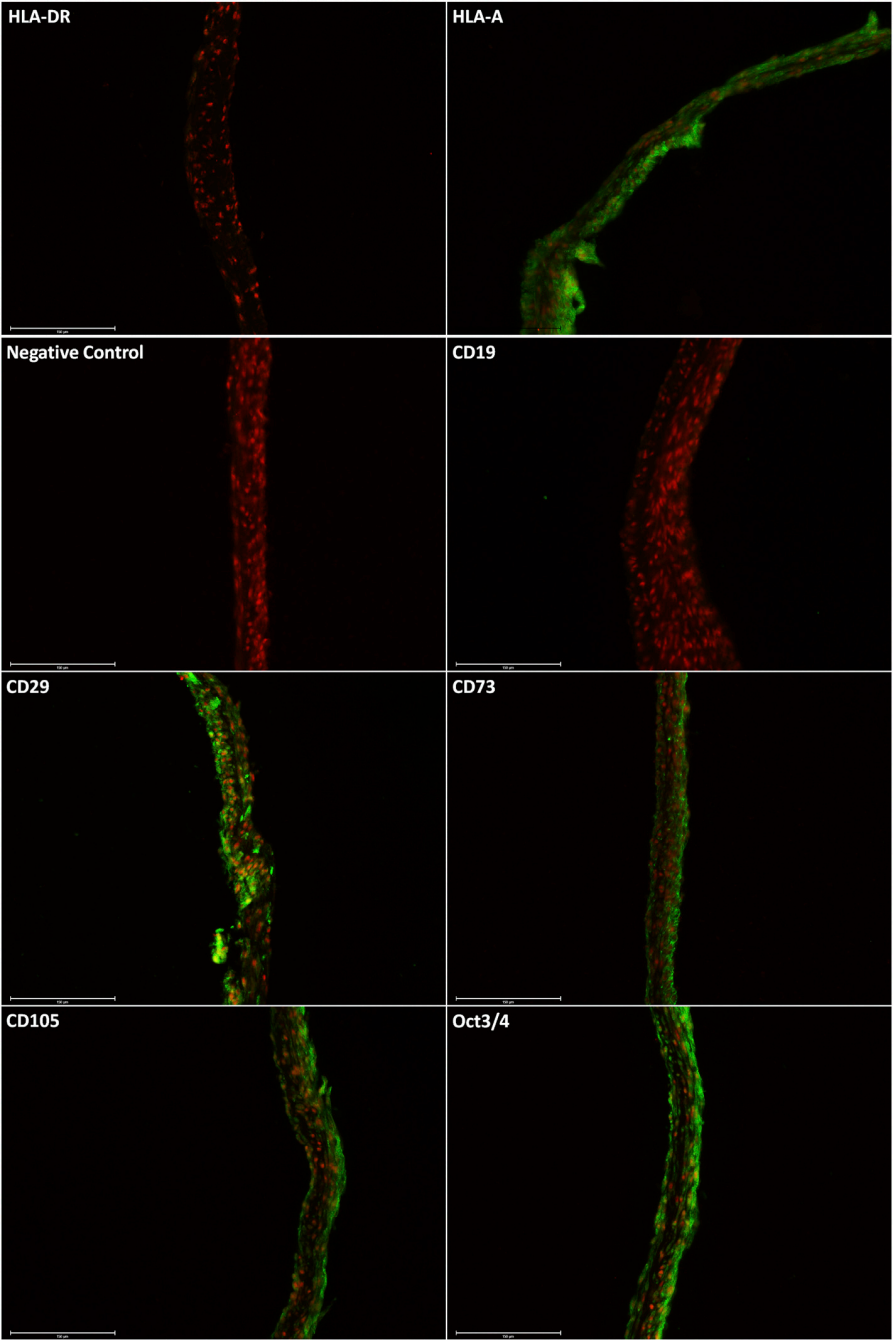
Immunostaining shows the positive expression of HLA-A, CD29, CD73, CD105, and Oct3/4. The proteins HLA-DR and CD19 were not detected. The scale bars are 150 µm.

Immunostaining of the harvested cell sheets was carried out by Alcian blue and Alizarin red dyes. In Figure 3, the expression of specific markers of ASCs was confirmed: HLA-A^+^, CD29^+^, CD73^+^, CD105^+^, Oct3/4^+^, HLA-DR^-^, and CD19^−^ (Figure 3) (Dominici et al., 2006).

**FIGURE 4:**
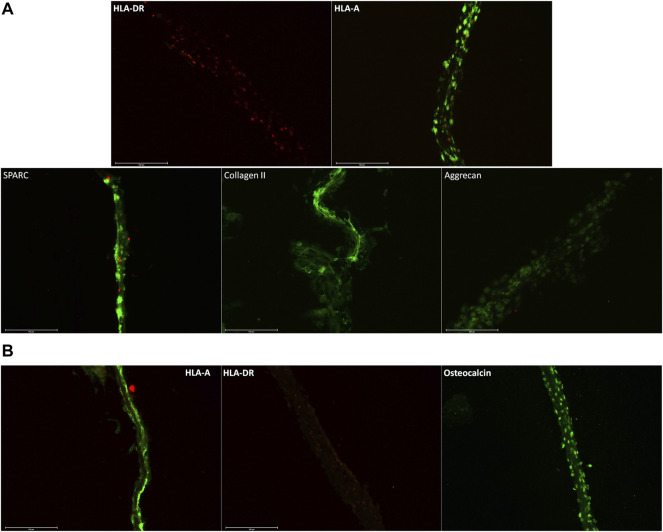
**(A)** Immunostaining of chondrocyte cell sheets, with HLA-A^+^, SPARC^+^, collagen II^+^, aggrecan^+^, and HLA-DR^-^. SPARC, collagen II, and aggrecan being specific markers of cartilage. **(B)** Immunostaining on osteoblast cell sheets with osteocalcin, a specific marker of osteoblast. Scale bars are 150 µm.

In Figure 4, we confirmed the expression of specific markers of chondrocytes and osteoblast cell sheets. In Figure 4A, chondrocyte cell sheets expressed HLA-A and secreted protein acidic and rich in cysteine (SPARC) (Aeschlimann et al., 1995), collagen II (Lian et al., 2019), and aggrecan (PMID: 11942407) (Kiani et al., 2002). In Figure 4B, osteoblast cell sheets expressed HLA-A and osteocalcin (Manolagas, 2020). For both types of cell sheets, HLA-DR was not detected, which will allow allogeneic transplantation with a lower risk of immune rejection.

**FIGURE 5:**
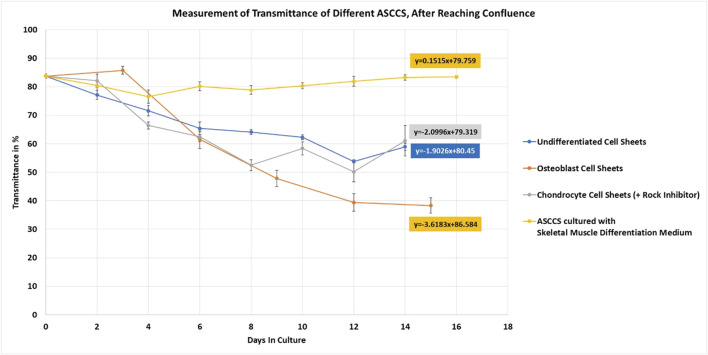
Measurement of undifferentiated, osteoblast, and chondrocyte cell sheets. ASCCSs cultured with skeletal muscle differentiation culture media were used as a control to show the unchanged transmittance over time. Number of days in culture is the number of days after the cells reached confluence (day 4) (number of cell sheets measured per point *n* = 5, except for days 0, *n* = 10).

Once the protocol to engineer different types of ASCCSs was established, the transmittance of the cell sheets was measured until harvesting (Figure 5). ASCCSs cultured with skeletal muscle differentiation culture media did not grow over time (Figure 1). The cell density was stable for 16 days, and it is concordant with the stability of the transmittance (Figure 5, yellow line). It was not possible to harvest those cells because of the low cell density, which plays a role in the cell–cell connection, observed in the other cell sheets. The transmittance of undifferentiated cell sheets and chondrocyte cell sheets was similar, even if the chondrocyte cell sheet transmittance was lower (reaching 50%), when undifferentiated ASCCSs reached 53%. One important morphological cell difference between both cell sheets is that the chondrocyte cells are round and their number increases over time, whereas the cells from the undifferentiated cell sheets maintain some of their fibroblastic shape and form harmonious “waves” of cells.

On day 14, the transmittance was similar along with the macro morphology of the cell sheets. Undifferentiated cell sheets can be harvested once the transmittance is lower than 75% (data not shown). However, to compare the transmittance over time, among the four types of cell sheets, undifferentiated ASCCSs were maintained in the culture. The slope of the curves is very similar: -1.9 for the undifferentiated ASCCSs and -2 for the chondrocyte cell sheets (Figure 5 blue and gray lines). The transmittance of the osteoblast cell sheets was interesting because the transmittance of the cell sheets always increased during the first three days of cell culture. This increase is because the cells are contracting and getting smaller (Figure 5, day 3 of differentiation), as a morphological transition (Figure 5, orange line). However, soon after this transmittance increase, the transmittance decreased much faster than that of the undifferentiated and chondrocyte cell sheets. The slope of the curve is -3.6, and it could be explained by the accelerated accumulation of calcium in the cells, which is correlated with cell sheet opacity increase (Figures 2, 5). The transmittance reached 38% on the day of harvesting.

**FIGURE 6:**
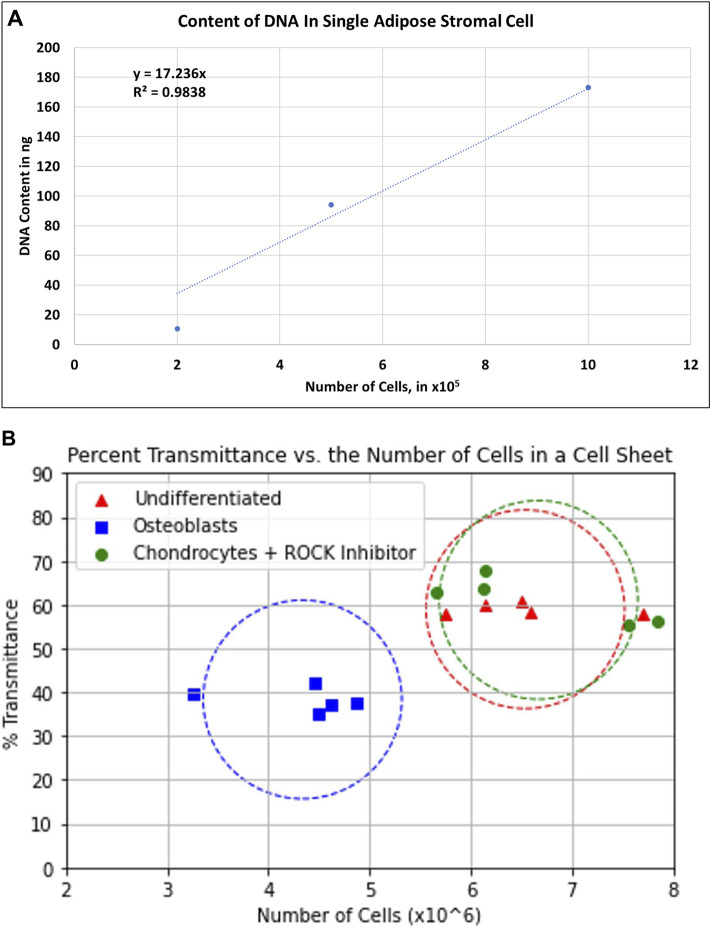
**(A)** Standard curve of total DNA content per number of single adipose stromal cells. The equation y = 17.236x was used to estimate the number of cells from each cell sheet. **(B)** Graph showing the correlation between the number of cells per type of cell sheet and the transmittance.

Undifferentiated chondrocyte and osteoblast cell sheets were harvested easily, using a CellShifter. These cell sheets can be transplanted onto the patients. One major piece of information to provide to the FDA is the number of cells transplanted onto the patient. To determine the exact number of cells, the whole cell sheets must be dissociated with enzymes resulting in that the cell sheets can no longer be transplanted. Using a relation between the transmittance and the total DNA content, the estimation of cell number per cell sheet is possible. In Figure 6, the relation between those two values is shown. Using a standard where the number of ASCs corresponds to the quantity of isolated DNA; the number of cells per cell sheet was determined after the isolation of the total cell sheet DNA. As the transmittance of each cell sheet was recorded independently, we could correlate the number of cells per cell sheet with the transmittance (Figure 6). Osteoblast cell sheets had the lowest number of cells, despite having the lowest transmittance. This is correlated with the possibility of harvesting the cell sheets where the osteoblast cell sheets seem thinner than the other two ones. The undifferentiated and chondrocyte cell sheets have a higher number of cells and transmittance values. The center of the circles is calculated based on the transmittance average of the transmittance and the average of the cell number. The objective is that with the measured transmittance of the cell sheets cultured in specific culture media placed in these circles will help estimate the number of cells per cell sheet before transplantation, using a non-invasive and safe methodology.

## Discussion

Numerous studies reported the beneficial effects of using MSCs in the treatment of cartilage (Ma et al., 2018), graft-versus-host disease (Zhao et al., 2019), ischemia (Oliva, 2019), myocardial infarction (Luo et al., 2017), and sclerosis (Dudek et al., 2020). In this study, osteoblast and chondrocyte cell sheets were engineered, which can be harvested and transplanted in the required areas (Tuan et al., 2013; Richardson et al., 2016; Oryan et al., 2017; Yorukoglu et al., 2017; Abdel Meguid et al., 2018; Egido-Moreno et al., 2021). The use of chemically defined culture media, in the absence of serum, is strongly recommended by the FDA and can also aid in not having to change the protocols once the product needs to be produced following the FDA guidelines. We decided to focus on chemically defined serum-free culture media that could be used for clinical trials. These two criteria are two major factors that play a role in terms of reproducibility and can facilitate the transition to clinical studies. Undifferentiated osteoblast and chondrocyte cell sheets were engineered with success by overgrowing the cells and by inducing the differentiation once the ASCs reached confluence. Our approach is easy to reproduce. The same culture media can be used throughout the differentiation time, whereas other studies have shown that different culture media must be used to obtain MSC differentiation (Park and Cho, 2010). The ASC cell sheets expressed specific markers (Figures 2–4), and the absence of HLA-DR will help build cell sheet banks for allogeneic transplantation. As mentioned, cell sheets have other advantages such as the increase not only in the production of cytokines but also the production of proteins involved in the cell–cell connection. Okano’s group reported that compared with a 2D monolayer umbilical cord mesenchymal stem cell, 3D cell sheets produced more β-catenin, connexin 43, integrin β1, and laminin (Bou-Ghannam et al., 2021). The production of these proteins explains why the cell sheets are strong enough to be harvested and lifted mechanically, without using enzymes or a thermo-responsive surface. As reported by our group in this study, the difference in the harvested cell sheet physical properties was noticed, but we were not able to translate them into numbers or graphs. We understand that it is necessary to develop new tools and experiments to understand better the cell sheet physical properties as explained in this excellent review (Efremov et al., 2021). Different physical properties could be studied, such as how the cell sheets can be stretched in one or two directions (Homma et al., 2020; Huang et al., 2021), the nanoindentation (Qian and Zhao, 2018), or optical properties (Ochiai et al., 2021). Shimizu’s group reported that detached and stretched cardiomyocyte cell sheets from a temperature-responsive surface were longer, but that the cardiomyocyte cells had a unidirectional alignment. The cells in the control cell sheets were randomly aligned. For such a methodology, the cell sheets must be detached from the support and can be transplanted or seeded again on a cell culture dish for further studies (Homma et al., 2020). Nanoindentation could be used directly on the 3D cell sheets to determine the resistance of the cell sheets to forces and could help determine if the cell sheets are strong enough for harvesting or strong enough for medical purposes. Such techniques could be used to increase our knowledge about 3D cell sheets. Nanoindentation is a non-invasive approach that was not used to the best of our knowledge. We decided to use another non-invasive approach to study the 3D engineered cell sheets. A device, developed by our group, was used to study the changes in cell sheet transmittance over time and during the differentiation (Ochiai et al., 2021). We reported that the transmittance of the three types of cell sheets decreased and that once the transmittance reached a certain value, it was possible to mechanically harvest the undifferentiated cell sheets (Ochiai et al., 2021). However, the value of the transmittance does not reflect the strength of the cell sheets. Chondrocyte and undifferentiated cell sheets were strong and easy to harvest. On the other hand, the transmittance of the osteoblast cell sheets was the lowest, but the harvesting of the cell sheets was more delicate; cell sheets could break easily compared with the undifferentiated and chondrocyte cell sheets. The use of nanoindentation will be a very good tool to determine how resistant the cell sheets are and correlate their strength with the outcome after transplantation. Transmittance values can help in following the differentiation of the cell sheets over time, but they also can help in estimating the number of cells per cell sheet CAR-T cells are approved by the FDA to treat certain cancers. CAR-T are single cells, and it is easy to determine how many cells are injected per patient, but it is impossible to know how many cells in a cell sheet are transplanted (Kochenderfer et al., 2015; Neelapu et al., 2017; Schmidts and Maus, 2018). The only way to know exactly how many cells compose a cell sheet is to digest it with enzyme, but this will make it useless for transplantation. We have developed a device that can translate the level of cell sheet transmittance to estimate the time of cell sheet harvesting (Ochiai et al., 2021). In addition, by measuring the transmittance, the day of harvesting, and the number of cells isolated from the cell sheets, we will be able to estimate the number of cells per type of cell sheet, cultured under specific conditions.

Engineering chondrocyte cell sheets is more difficult than engineering undifferentiated and osteoblast cell sheets. For the chondrocyte cell sheet engineering, using the MesenCult-ACF culture media by itself, the success rate in engineering chondrocyte cell sheets was 11% (3/27). Another group reported, which we also noticed, that during the chondrocyte differentiation, cell sheets were spontaneously detaching from the edge and detaching completely from the bottom of the cell culture dish, at early stages (less than 6–8 days from the starting differentiation day) (data not shown) (Thorp et al., 2020). Not being satisfied by the low rate of success in engineering chondrocyte cell sheets, we noticed that the cause of the complete detachment of the cell sheets was due to forces exercised at the edge of the cell sheets. The cytoskeleton plays a role in this spontaneous detachment. Cytoskeleton proteins are involved in the organization of the cell–cell connection and the cell–extracellular matrix connection. It was reported that cells exert a certain tension over the surface when they are in contact with the extracellular matrix (ECM) (Ganz et al., 2006). In a publication, Schiller et al demonstrated that the pathway involving ROCK mediates the forces at the edges of the cells; accordingly, the same pathway could be the cause of the spontaneous cell sheet detachment. Manually cutting the edge of the cell sheets from the cell culture dishes, once we noticed that the cell sheets were detaching slowly from the edges, improved the production of chondrogenic cell sheets (data not shown). However, cutting the edges is not practical for the GMP cell sheet manufacturing stage and could increase the risk of cell sheet damage and contamination. Based on the cited literature, an ROCK inhibitor, such as Y27632, was used to improve the chondrogenic differentiation of MSCs. The ROCK inhibitor was shown to decrease the cytoskeleton tension of MSCs and increased the chondrogenic differentiation of the MSCs (Wang et al., 2018). When the ASCs reached confluence, they were cultured with the chondrogenic differentiation culture media, complete with Y27632 (10 µM final concentration) from the first to the last day of differentiation. It is important to note that when the ROCK inhibitor was added to MesenCult-ACF at 10 µM, the success rate was 100% (45/45), confirming the idea that the ROCK inhibitor improved the success rate of engineering chondrocyte cell sheets. No additional stimuli were used to produce the chondrocyte cell sheets such as vibration, compression, gravity, magnetic field (Zhang et al., 2015; Uddin et al., 2016; Hou et al., 2020; Li et al., 2020), and stacking (Zhou et al., 2015; Enomoto et al., 2016).

We are aware of the limitations of this study. Many more cell sheets should be engineered to obtain a better estimation of the number of cells per cell sheet, in correlation with the transmittance. In addition, the reported data are based on our cell culture media and the cells we have used, and different data could be obtained if the quality of the cells or culture media is different. However, this device can provide valuable information in terms of physical properties, maturity of the cell sheets, and the cell sheet posology using a non-invasive approach. In addition, animal studies will be required to evaluate the xenotransplantation of the cell sheets in terms of regenerative properties and confirm their hypo-immunogenicity.

## Conclusion

We succeeded in engineering three types of cell sheets, using a xeno/serum-free culture media, and were able to harvest them. Undifferentiated ASCCSs maintained the expression of ASC markers. Chondrocyte cell sheets expressed specific markers (SPARC, collagen II, and aggrecan) along with the osteoblast cell sheets (osteocalcin). Based on the transmittance measured on the harvesting day and a standard DNA quantification from a single ASC, we can estimate the number of cells per cell sheet and per type of cell sheets. Using an affordable non-invasive method, this device could be used in translational regenerative medicine.

## Data Availability

The original contributions presented in the study are included in the article/Supplementary Material, further inquiries can be directed to the corresponding author.
